# Study on the correlation between preoperative inflammatory indexes and adhesional perinephric fat before laparoscopic partial nephrectomy

**DOI:** 10.1186/s12894-021-00940-2

**Published:** 2021-12-10

**Authors:** Teng Ma, Lin Cong, Qianli Ma, Zhaoqin Huang, Qianqian Hua, Xiaojiao Li, Ximing Wang, Yunchao Chen

**Affiliations:** 1 Department of Radiology, Shandong Provincial Hospital Affiliated to Shandong First Medical University, No.324, Jingwu Road, Jinan, 250021 Shandong China; 2 Department of Medical Imaging Interventional Therapy, Shandong Provincial Hospital Affiliated to Shandong First Medical University, Jinan, 250021 Shandong China; 3 Department of Radiology, Taian City Central Hospital, Taian, 271000 Shandong China

**Keywords:** Preoperative composite inflammatory index, Adhesional perinephric fat, Laparoscopic partial nephrectomy, Renal cell carcinoma

## Abstract

**Objective:**

This study was aimed to evaluate the effect of preoperative composite inflammatory index on adhesional perinephric fat (APF), providing a help for preoperative risk assessment of laparoscopic partial nephrectomy (LPN) in patients with renal cell carcinoma.

**Materials and methods:**

A retrospective study was conducted on 231 patients with renal cell carcinoma, who underwent laparoscopic partial nephrectomy. They were divided into two groups according to whether there was APF during operation. Relevant clinical data, laboratory parameters and imaging examination were obtained before operation to calculate the composite inflammatory index and MAP score. The composite inflammatory index was divided into high value group and low value group by ROC curve method. The related predictive factors of APF were analyzed by logistic regression method.

**Results:**

The APF was found in 105 patients (45.5%). In multivariate analysis, systemic immune inflammation index (SII) (high/low), MAP score, tumor size and perirenal fat thickness were independent predictors of APF. The operation time of patients with APF was longer, and the difference of blood loss was not statistically significant.

**Conclusion:**

SII is an independent predictor of APF before laparoscopic partial nephrectomy.

*Trial registration* ChiCTR, ChiCTR2100045944. Registered 30 April 2021—Retrospectively registered, http://www.chictr.org.cn/showproj.aspx?proj=125703.

## Introduction

Partial nephrectomy is the preferred choice for cT1a renal tumors [1], including open partial nephrectomy (OPN), laparoscopic partial nephrectomy (LPN) and robotic partial nephrectomy (RPN). Preoperative evaluation of the feasibility and difficulty of partial nephrectomy is a hot topic in clinical research. In addition to the anatomic characteristics of the tumor itself and the thickness of perirenal fat affecting partial nephrectomy, adhesional perinephric fat (APF) is another important affecting factor [2, 3].

APF is defined as the inflammatory tissue adhering around the kidney [4], which makes the dissociation of the kidney and the exposure of tumor more difficult, increases the difficulty of operation, prolongs the operation time, and may lead to separation bleeding and renal capsule stripping [5]. The mechanism of APF is complex, and chronic systemic inflammation may be one of its potential causes [6, 7]. Studies have found that inflammatory immune response is involved in the occurrence and development of tumor, and many inflammatory indicators have been confirmed as high-risk factors for tumor prognosis [8–10]. In recent years, a number of new composite inflammatory indicators have been used to evaluate the body's inflammatory response, including neutrophil to lymphocyte ratio (NLR), platelet to lymphocyte ratio (PLR), monocyte to lymphocyte ratio (MLR), prognostic nutritional index (PNI), systemic inflammation response index (SIRI), systemic immune inflammation index (SII) and so on. There is no relevant research report on the correlation between the adhesion of fat around the kidney and the above inflammatory indexes.

At present, image and image-based scoring systems are mainly used to evaluate the situation of peripheral fat adhesion. It has been reported that MAP score and perirenal fat thickness could be used as independent predictors of APF [6, 11]. However, the correlation between APF evaluation and composite inflammatory indicators has not been reported.

This study retrospectively analyzed the clinical data of patients with renal cell carcinoma who underwent partial nephrectomy in our hospital in recent years, to explore the correlation between APF and preoperative composite inflammatory indicators.

## Materials and methods

### Participants

**Fig. 1:**
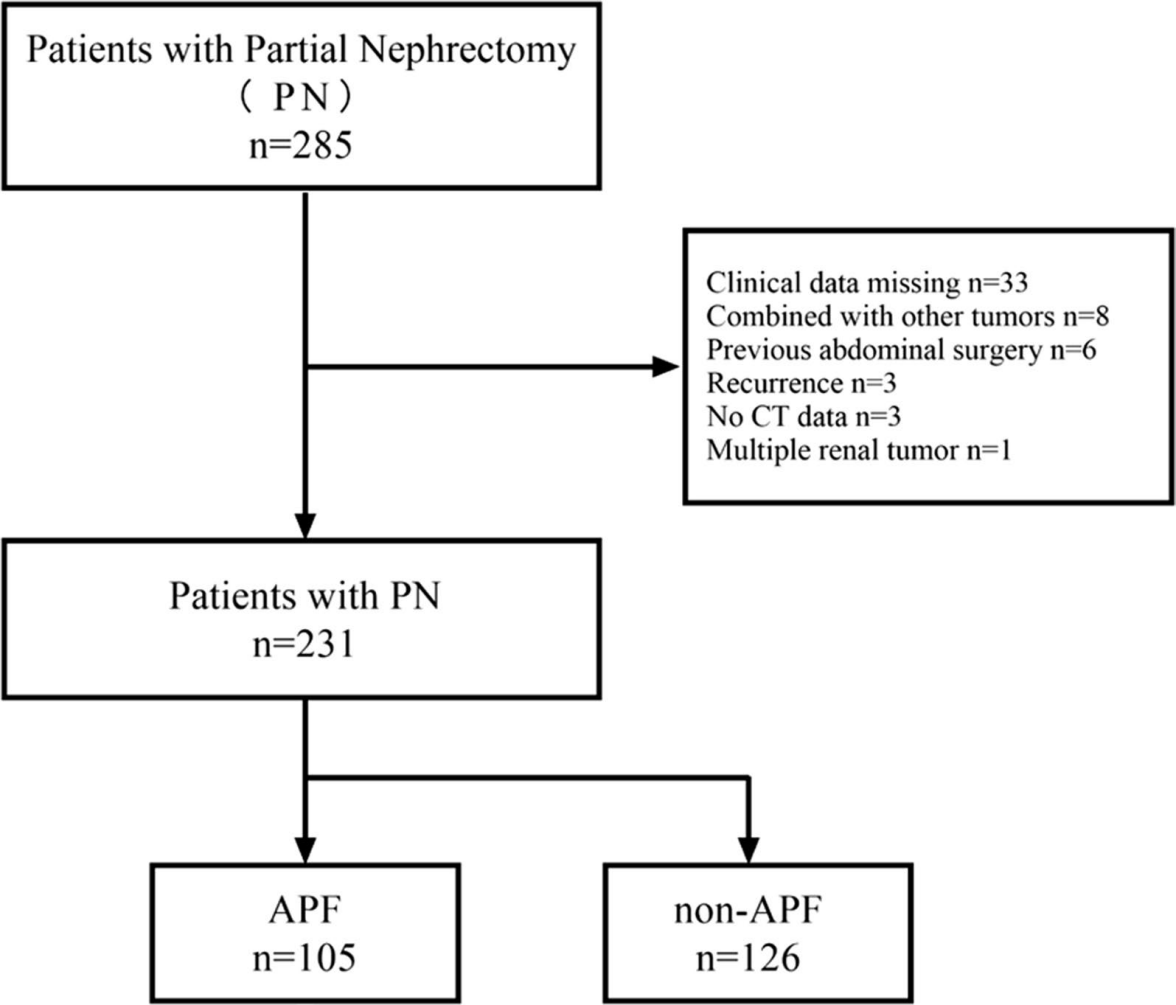
Patient enrolment process

The data of 285 patients with renal cell carcinoma who underwent laparoscopic partial nephrectomy in our hospital from January 2016 to December 2020 were retrospectively analyzed. Fifty-four patients were excluded because of incomplete clinical data. A total of 231 patients were included in the study (Fig. 1), including 156 males and 75 females, with the median age of 55 (IQR 46–63). Laparoscopic partial nephrectomies were all performed by four surgeons with more than five years of surgical experience. According to the operation record by five surgeons with 11 years, 12 years, 12 years, 14 years, and 15 years experiences, respectively, the patients were divided into adhesion group and non-adhesion group according to whether there was APF (perinephric fat showed inflammatory changes and adhesion with renal capsule, renal tumors are difficult to expose). The radiological evaluation of images was independently performed by a radiologist and one of the surgeons. If the score differed between the two observers, a consensus was reached together after discussion.

Inclusive criteria: (1) single renal tumor with normal contralateral renal structure; (2) preoperative computed tomography; (3) no previous history of renal surgery and abdominal surgery; (4) no previous history of malignant tumor; (5) exclusion of preoperative acute urinary tract infection, pulmonary infection, autoimmune diseases and hematological diseases.

### Data collection

The general data of patients were collected, including age, gender, past history and personal history.

Operation related data were collected, including operation time, estimated blood loss and adhesion.

Image related data were collected, including R.E.N.A.L score, MAP score and perirenal fat thickness obtaining from preoperative computed tomography. Perinephric fat thickness was evaluated selecting the CT slice of interest at the level of renal hilum. Posterior perirenal fat thickness was measured from the kidney capsule to the posterior abdominal wall. Perinephric fat stranding was defined as follows: absent (0 points), moderate (2 points), severe (3 points). The MAP score was calculated for each subject using measurements of postrenal fat thickness and perirenal fat chain hypothesized by Davidiuk et al. [6].

The routine examination results of peripheral venous blood within 3 days before operation were collected, including neutrophil count, lymphocyte count, monocyte count, platelet count, CRP and eGFR.

The results of SII, SIRI and PNI were calculated according to the following formula.

$${\text{SII}} = {\text{platelet count }}\left( {{10}^{{9}} /{\text{L}}} \right) \times {\text{neutrophil count }}\left( {{10}^{{9}} /{\text{L}}} \right)/{\text{lymphocyte count }}\left( {{1}0^{{9}} /{\text{L}}} \right)$$

$${\text{SIRI}} = {\text{monocyte count }}\left( {{10}^{{9}} /{\text{L}}} \right) \times {\text{neutrophil count }}\left( {{10}^{{9}} /{\text{L}}} \right)/{\text{lymphocyte count }}\left( {{1}0^{{9}} /{\text{L}}} \right)$$

$${\text{PNI}} = {{10}} \times {\text{serum albumin}}\left( {{\text{g}}/{\text{dl}}} \right) + 0.00{\text{5}} \times {\text{lymphocyte count}}\left( {{\text{mm}}^{{3}} } \right)$$

### Statistical method

All analyses were performed with SPSS Statistics, version 23.0 (IBM Corp., Armonk, NY, USA). Descriptive statistics were obtained reporting medians (and interquartile ranges, IQR) for continuous variables, and frequencies and proportions for categorical variables. Univariate and multivariate logistic regression analysis were used to predict the risk factors of APF. *P* < 0.05 was considered statistically significant. Independent sample t test was used to compare the measurement data between groups, and chi square test was used to compare the count data between groups.

## Results

### General information

In this study, 231 patients were collected, including 156 males and 75 females, with the median age of 55 (IQR 46–63). Among them, 45.0% had a history of hypertension, 37.7% had dyslipidemia, 28.1% had a history of smoking, 17.7% had a history of diabetes, and 7.8% had cardiovascular disease (Table 1).

**Table 1:** General information

	All	APF	Non-APF	*P*
Number (%)	231 (100)	105 (45.5)	126 (54.5)	
Age, median (IQR)	55 (46–63)	56 (47–64)	54 (45–60)	0.024
Sex, n (%)				
Male	156 (67.5)	76 (72.4)	80 (63.5)	
Female	75 (32.5)	29 (27.6)	46 (36.5)	
Cardiovascular disease, n (%)	18 (7.8)	8 (7.6)	10 (7.9)	0.929
Hypertension, n (%)	104 (45.0)	59 (56.2)	45 (35.7)	0.002
Diabetes, n (%)	41 (17.7)	18 (17.1)	23 (18.3)	0.826
Dyslipidemia, n (%)	87 (37.7)	39 (37.1)	48 (38.1)	0.882
Smoking, n (%)	65 (28.1)	33 (31.4)	32 (25.4)	0.310
CRP, median (IQR)	1.24 (0.68–2.38)	1.44 (0.79–2.93)	1.14 (0.63–2.19)	0.425
eGFR, median (IQR)	104 (94–111)	104 (93–111)	104 (96–111)	0.425
Creatinine, median (IQR)	65.1 (56.1–74.6)	67.1 (57.3–75.5)	64.1 (54.8–74.5)	0.461
Albumin, mean median (IQR)	42.3 (40.4–44.5)	41.9 (40.0–44.4)	42.8 (40.8–44.7)	0.056
Tumor size, median (IQR)	2.8 (2.0–3.8)	3.0 (2.5–4.2)	2.5 (2.0–3.4)	< 0.001
Location, n (%)				0.754
Left	106 (45.9)	47 (44.8)	59 (46.8)	
Right	125 (54.1)	58 (55.2)	67 (53.2)	
Pathology, n (%)				0.315
Clear cell carcinoma	206 (89.2)	96 (91.4)	110 (87.3)	
Non-clear cell carcinoma	25 (10.8)	9 (8.6)	16 (12.7)	
R.E.N.A.L score, n (%)				0.068
4–6	63 (27.3)	21 (20.0)	42 (33.3)	
7–9	150 (64.9)	76 (72.4)	74 (58.8)	
10–12	18 (7.8)	8 (7.6)	10 (7.9)	
MAP score, median (IQR)	2 (1–3)	3 (2–4)	1 (0–2)	< 0.001
Perirenal fat thickness, median (IQR)	1.1 (0.6–1.7)	1.4 (0.9–2.1)	0.9 (0.5–1.2)	< 0.001
Blood loss, median (IQR)	50 (30–100)	50 (30–100)	30 (30–50)	0.205
Operation time, median (IQR)	130 (110–165)	150 (120–180)	120 (99–150)	< 0.001

The results showed that there were significant difference in age (*P* = 0.024), history of hypertension (*P* = 0.002), tumor size (*P* < 0.001), MAP score (*P* < 0.001) and perirenal fat thickness (*P* < 0.001) between patients with APF and patients with non-APF using univariate analysis. Furthermore, the operation time of patients with APF was longer than patients with non-APF (150 min vs 120 min, *P* < 0.001). For blood loss, there was no significant difference between groups (50 ml vs 30 ml, *P* = 0.205).

### Adhesional perinephric fat

**Fig. 2:**
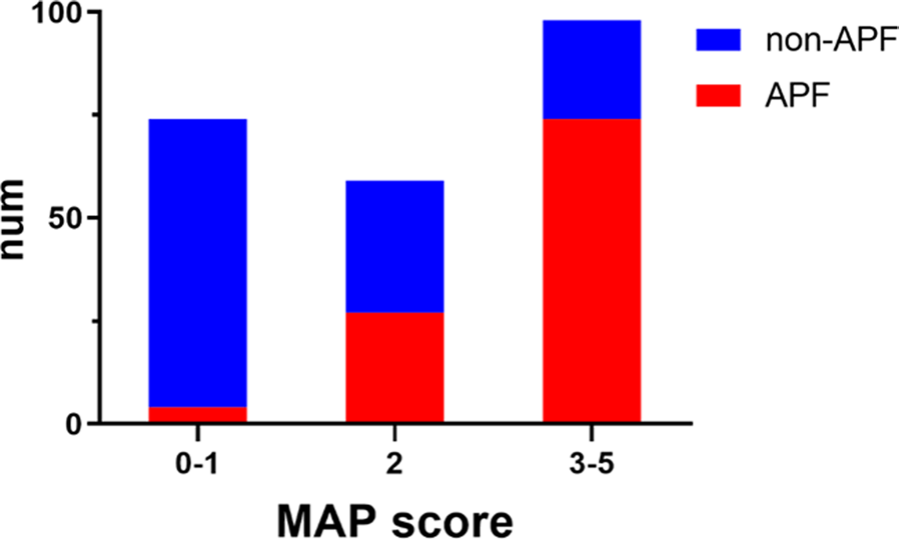
MAP score distribution in patients

The median MAP score was 2 in patients. Patients with APF was observed in 105 patients (45.5%, 105/231), and the corresponding MAP score was higher than patients with non-APF. Among patients with APF, there were 4 patients (5.4%, 4/74) with MAP score of 0–1, 27 patients (45.8% 27/59) with MAP score of 2, and 74 patients (75.5% 74/98) with MAP score of 3–5 (Fig. 2).

### Analysis of inflammatory indexes

The ROC curves of inflammatory indexes were obtained according to the presence or absence of APF. According to the Youden index, the optimal cut-off values of PNI, MLR, PLR, NLR, Siri and SII were 54.9, 0.25, 119.43, 2.14, 0.87 and 523.65, respectively. All patients were divided into high value group and low value group. There were significant differences between groups in NLR (*P* = 0.002), MLR (*P* = 0.007), SIRI (*P* < 0.001) and SII (*P* < 0.001) (Table 2).

**Table 2:** Analysis of inflammatory index between groups

	APF	Non-APF	*P*
PNI			
Low	82	86	0.094
High	23	40	
PLR			
Low	37	53	0.290
High	68	73	
NLR			
Low	55	91	0.002
High	50	35	
MLR			
Low	48	80	0.007
High	57	46	
SIRI			
Low	46	90	< 0.001
High	59	36	
SII			
Low	53	95	< 0.001
High	52	31	

The independent predictor of APF was analyzed using multivariate analysis (Table 3). The results showed that tumor size (OR = 1.453, *P* = 0.009), MAP score (OR = 4.545, *P* < 0.001), perirenal fat thickness (OR = 0.503, *P* = 0.043), SII (OR = 0.278, *P* = 0.018) were independent predictors for patients with APF.

**Table 3:** Multivariate logistic analysis

Index	Single factor analysis		Multivariate analysis	
	OR (95% CI)	*P*	OR (95% CI)	*P*
Age	1.027 (1.003–1.050)	0.025	–	–
Hypertension	2.309 (1.358–3.924)	0.002	–	–
Tumor size	1.512 (1.205–1.897)	< 0.001	1.453 (1.100–1.023)	0.009
MAP	3.173 (2.346–4.293)	< 0.001	4.545 (2.841–7.273)	< 0.001
Perirenal fat thickness	2.622 (1.770–3.883)	< 0.001	0.503 (0.258–0.978)	0.043
NLR	0.423 (0.245–0.731)	0.002	–	–
MLR	0.484 (0.286–0.821)	0.007	–	–
SIRI	0.312 (0.181–0.538)	< 0.001	–	–
SII	0.333 (0.190–0.581)	< 0.001	0.278 (0.097–0.800)	0.018

## Discussion

With the development of laparoscopy and robot assisted technology, nephron sparing partial nephrectomy has become the preferred treatment for early small renal cell carcinoma [1, 12]. Tumor itself and patient specific variables can increase the complexity of partial nephrectomy [5]. Patient factors refer to the influence of their own physical condition on the operation, including the presence or absence of accessory renal artery and APF, among which APF is the most important factor [4, 13]. APF can cause a series of intraoperative complications. Mibervini et al. studied 1055 patients who underwent PN and found that patients experiencing intraoperative complications had a significantly higher rate of overall postoperative complications (41.6%), surgical postoperative complications (29.2%), Clavien 2 surgical postoperative complications (14.6%) and a significantly longer length of stay (8 days) [14]. Efforts should be made to minimize the risk of intraoperative complications during PN. A recent study showed that assessing the effects of perinephric fat surface density in patients treated with OPN and RPN could help surgeons select the best approach and reduce perioperative complications [15]. However, the mechanism of APF is unclear, which may be related to fibrosis, autoimmunity and inflammatory reaction [3, 16]. In order to further clarify the relationship between the occurrence of APF and systemic inflammatory response in renal cancer patients, 231 patients were collected.

In this study, patients were divided into high value group and low value group according to the best cut-off value of each inflammatory factor. The best cut-off value was obtained from the ROC curve in patients with APF or non-APF. The results showed that there were significant differences in NLR (*P* = 0.002), MLR (*P* = 0.007), SIRI (*P* < 0.001) and SII (*P* < 0.001) between groups. Furthermore, the SII was an independent predictors for patients with APF (*P* = 0.018).

The levels of neutrophils, lymphocytes and monocytes in peripheral blood reflect the immune function of human peripheral blood and the tumor microenvironment. Neutrophils come from bone marrow, and mature neutrophils are distributed in peripheral blood, which main functions are phagocytosis and killing bacteria. The increase of neutrophils in tumor patients may be due to the secretory function of tumor tissue, which can produce a variety of cytokines to promote bone marrow growth, such as granulocyte colony-stimulating factor (G-CSF) or granulocyte macrophage colony-stimulating factor (GM-CSF) [17, 18]. These growth factors can increase the number of neutrophils in peripheral blood. The increase of neutrophils inhibits the immune activity of lymphocytes, resulting in the decrease of lymphocytes and the decrease of immune function [19]. Under the action of inflammatory chemokines, monocytes in peripheral blood migrate around tumor tissue to form tumor associated macrophages (TAM), which participate in inflammatory immune response and tumor growth [20, 21]. Cytokines produced by systemic inflammatory reaction can also increase the number of platelets, release platelet-derived growth factor, platelet-reactive protein and platelet factor-4, which promote tumor proliferation, invasion and angiogenesis [22, 23]. The increase of neutrophils, platelets and monocytes in the blood often represents the aggravation of inflammation, while the decrease of lymphocyte is the decrease of anti-tumor immune response. The composite inflammatory index composed of inflammatory cells can reflect the relative changes of different blood components, which reflect the host immunity and inflammation more comprehensively compared with single inflammatory index. PLR, MLR and NLR have been proved to be potential prognostic indicators for a variety of malignant tumors [24–26]. SII is a new inflammation index based on neutrophil, platelet and lymphocyte count, which has been proved to be a negative prognostic indicator of small cell lung cancer, hepatocellular carcinoma, esophageal squamous cell carcinoma, and esophagogastric junction tumor [27, 28].

In this study, there were significant differences between high value group and low value group in NLR, MLR, SIRI and SII, which indicated that there was a correlation between APF and systemic inflammatory response. The possible mechanism is that the systemic chronic inflammatory pathway is activated to trigger tissue adhesion. For example, the activated cytokines including interleukin-6 and tumor necrosis factor (TNF-α), chemokines and plasminogen activator inhibitor type 1 (PAI-1) causing the decrease of fibrinolytic activity [29–31]. When this reaction affects the perirenal fat, it will cause fiber adhesion between the fat and renal capsule, leading to the formation of APF, which is manifested as cord like and nodular increased density foci in the perirenal space on CT imaging. Our results were similar to Dariane et al. [32] found that low-level chronic inflammation may eventually lead to renal radiofibrosis.

On the other hand, the role of local inflammatory microenvironment produced by malignant tumor could not be ignored. In this study, the incidence of APF in 231 patients with renal cell carcinoma was 45.5% (105/231), which was close to the results of previous studies [32]. Inflammatory cells, cytokines and chemokines affect tumor growth, differentiation, diffusion and metastasis [33]. Similarly, tumor cells can also react on inflammatory cells. Tumor cells can induce the enhancement of platelet activity and promote the release of platelet granule contents, such as 5-hydroxytryptamine and ADP [34]. Druce et al. [35] found 5-hydroxytryptamine is an important mediator of local and distal fibrosis in small intestinal carcinoid. Therefore, we infer that renal tumor may also indirectly lead to the saponification and adhesion of perirenal fat through the two-way effect of inflammatory microenvironment.

In our study, patients with APF were correlated with age, history of hypertension, tumor size, MAP score and perirenal fat thickness, but not with gender and history of diabetes. Fabrizio et al. showed that metabolic syndrome was the only significant predictor of APF [15]. However, in our study, APF was associated with hypertension, but not with diabetes. The reasons for the differences may be as follows: our study sample size was small, and only patients undergoing LPN were included, but not patients undergoing OPN or RPN. However, it cannot be ruled out that there are a small number of patients who were previously treated for diabetes but did not provide an accurate history of the disease to us. The operation time of patients with APF was longer, but there was no significant difference in blood loss between the two groups. Multivariate logistic regression analysis showed that MAP score (OR = 4.545, *P* < 0.001), tumor size (OR = 1.453, *P* = 0.009), perirenal fat thickness (OR = 0.503, *P* = 0.043) and SII (or 0.278, *P* = 0.018) were independent predictors of perirenal fat adhesion. Previous studies have shown that perirenal fat thickness and MAP score have a strong correlation with APF [6, 36], but our study first reported that SII is one of the independent predictors of APF, further confirmed that systemic inflammatory immune response is involved in the formation of APF. Although MAP score is the strongest predictor of APF (OR = 4.545), we believe that in daily practice, the acquisition process of SII is simpler, and the measurement results are relatively more objective, and the economic cost is lower. Studies by Mari et al. showed that several clinical predictors were associated with postoperative complications [37]. In the future, we will study APF, SII and other factors to verify their influence on postoperative complications.

The limitations of this study are as follows: (1) limited to the retrospective case analysis, we could not further clarify the impact of the dynamic changes of inflammatory indicators on the formation of APF. (2) the critical value of each inflammatory index was determined using ROC curve according to the Youden index. However, there is no consensus on the selection of the critical value at this stage. Therefore, the bias caused by this choice may have a certain impact on the research results. (3) Due to the retrospective nature of the study, there are some missing clinical data of patients that further reduce the reliability of the results. (4) Since all patients underwent LPN, whether the findings of this study can be generalized to patients undergoing OPN or RPN needs further verification. (5) This study is a single center study with a relatively small sample size. Therefore, the exact results need to be confirmed by large-scale prospective multicenter studies in the future.

## Conclusions

This study showed that NLR, MLR, SIRI and SII were correlated with APF in patients with renal cell carcinoma, and SII was an independent predictor of APF. Because of the advantages of convenient detection, strong repeatability and low cost, inflammatory markers are expected to play a role in the early prediction of APF, which is helpful for surgeons to assess the operation related risks and make scientific and reasonable preoperative plans.

## Data Availability

The datasets during and/or analysed during the current study available from the corresponding author on reasonable request.
